# Preparation, characterization, identification, and antioxidant properties of fermented acaí (*Euterpe oleracea*)

**DOI:** 10.1002/fsn3.3274

**Published:** 2023-02-28

**Authors:** Wen‐Ying Liu, Xue Wang, Jie Ren, Cheng‐Dong Zheng, Han‐Shuo Wu, Fan‐Tong Meng, Kong Ling, Xiu‐Yu Qi, Ming Zhou, Yue Wang, Rui‐Zeng Gu, Lu‐Jia Han, Yong‐Jiu Zhang

**Affiliations:** ^1^ Engineering Laboratory for Agro Biomass Recycling & Valorizing College of Engineering, China Agricultural University Beijing People's Republic of China; ^2^ Heilongjiang Feihe Dairy Co., Ltd. Beijing People's Republic of China; ^3^ Beijing Engineering Research Center of Protein and Functional Peptides China National Research Institute of Food and Fermentation Industries Co., Ltd. Beijing People's Republic of China

**Keywords:** Acaí, acaí fermentation liquid, antioxidant capacity, scanning electron microscope, volatile aroma components

## Abstract

Fermentation technology was used to prepare the acaí (*Euterpe oleracea*) fermentation liquid. The optimal fermentation parameters included a strain ratio of *Lactobacillus paracasei*: *Leuconostoc mesenteroides*: *Lactobacillus plantarum* = 0.5:1:1.5, a fermentation time of 6 days, and a nitrogen source supplemental level of 2.5%. In optimal conditions, the ORAC value of the fermentation liquid reached the highest value of 273.28 ± 6.55 μmol/L Trolox, which was 55.85% higher than the raw liquid. In addition, the FRAP value of the acaí, as well as its scavenging ability of DPPH, hydroxyl, and ABTS free radicals, increased after fermentation. Furthermore, after fermentation treatment, the microstructure, basic physicochemical composition, amino acid composition, γ‐aminobutyric acid, a variety of volatile components, and so on have changed. Therefore, fermentation treatment can significantly improve the nutritional value and flavor of the acaí. This provides a theoretical basis for the comprehensive utilization of acaí.

## Graphical abstract

The physical, chemical, and volatile aroma components were analyzed and identified before and after acaí fermentation, showing an overall increase in these elements. Then, the oxygen radical absorbance capacity (ORAC) values, DPPH free radical scavenging capacity, hydroxyl free radical scavenging capacity, ABTS free radical scavenging capacity, and ferric reducing antioxidant power (FRAP) values were used as indicators to confirm that the antioxidant capacity of acaí fermentation liquid improved after fermentation. This provides a theoretical basis for the comprehensive utilization of acaí.

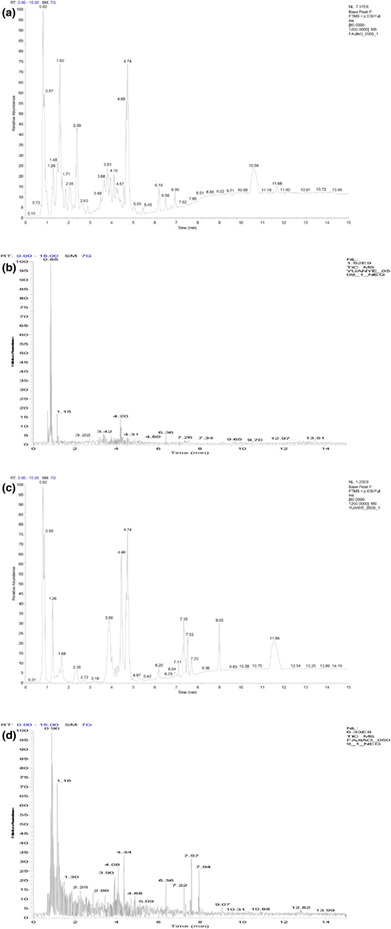

## 1 INTRODUCTION

Oxidative stress is closely related to the human body. When the human body is stimulated by exogenous or endogenous factors, the imbalance in the antioxidant system leads to oxidative stress, which can cause direct or indirect oxidative damage to biological macromolecules, such as DNA, proteins, and lipids, leading to physiological and pathological reactions (Hussain et al., 2003). Because free radicals have a single electron, they quickly attach to DNA, causing DNA strand breaks, DNA and protein cross‐links, and purine oxidation. Furthermore, free radicals destroy the peptides in the protein chain, facilitate protein cross‐linking, change spatial protein structures, and lead to protein inactivation. In addition, lipids containing a large amount of unsaturated fatty acids, such as phospholipids on cell membranes, are also vulnerable to free radicals, triggering a chain reaction of continuous free radical amplification. Malonaldehyde products can react to form lipofuscin, resulting in impaired biological macromolecule functionality (Fearon & Vogelstein, 1990). Sustained oxidative stress leads to chronic inflammation, which in turn contributes to most chronic diseases such as cancer, diabetes, cardiovascular, neurological, and lung diseases. In addition, higher intracellular oxidative stress aggravates the aging of the body (Reuter et al., 2010). Therefore, oxidative stress prevention is essential since it can cause cell damage and pathological reactions.

Acaí (*Euterpe oleracea*), also known as Brazil berry, has attracted considerable attention due to its strong antioxidant capacity and can be used as a functional food. It is a grape‐sized, purple‐black, protein‐rich berry that tastes like a combination of wild berries and chocolate (Lichtenthäler et al., 2005). Acaí is a major food source for local residents in Brazil, Peru, Colombia, and Suriname. Its juice is rich in bioflavonoids, anthocyanins, and other antioxidants and functional substances such as unsaturated fatty acids. These berries offer antioxidant (Alpert & Patricia, 2016) and anti‐inflammatory properties (Kang et al., 2010), lower blood lipids and blood pressure (de Souza et al., 2012; Silva et al., 2018), protect the liver (Carvalho et al., 2019), promotes bone health, and enhances pain relief (Brito et al., 2016; Jensen et al., 2011).

Due to higher health awareness, the quality of fermented food has improved in recent years, increasing in popularity with consumers. Fermentation involves using microbial technology for food production, and it is also the oldest method of producing and preserving food products. Fermentation retains active components in raw food, such as polysaccharides, dietary fiber, bioflavonoids, and other substances beneficial to the body. It also changes various raw nutritional components in food products to produce a unique flavor while decomposing factors unfavorable to human health, such as oligosaccharides and flatulence elements in beans. Furthermore, many metabolites produced via microbial metabolism can regulate the physiological functions of the body and inhibit the production of harmful substances. This study used acaí fruit pulp as raw material to examine the changes in the organic acid content, phenols, and other bioactive substances, chemical compositions, and antioxidant activity before and after fermentation. The fermentation technology was optimized to provide technical support and theoretical guidance for the production and development of fermented acaí products.

## 2 MATERIALS AND METHODS

### 2.1 Material

#### 2.1.1 Microorganisms

*Leuconostoc mesenteroides*, *Lactobacillus paracasei*, *Lactobacillus plantarum*, *Lactobacillus acidophilus*, *Lactobacillus salivarius*, *Lactobacillus delbrueckii* subsp. *bulgaricus*, *Streptococcus thermophilus*, *Lactobacillus Swiss*, *Lactobacillus fermentans*, and *Pediococcus pentosus* were purchased from the China Center of Industrial Culture Collection (CICC).

#### 2.1.2 Raw acaí liquid and reagents

The raw acaí liquid was provided by Heilongjiang Feihe Dairy Co., Ltd. Batch Number: 18102B0208231. The MRS broth medium was purchased from Beijing Luqiao Technology Co., Ltd., while the 2,2'‐Azobis(2‐methylpropionamidine)dihydrochloride (AAPH), fluorescein (fluorescent indicator), and Trolox (water‐soluble vitamin E) were obtained from the Sigma Company in the USA.

### 2.2 Preparation of the acaí fermentation broth

#### 2.2.1 Pretreatment of raw acaí liquid

Here, 1.5% wheat oligopeptide and 0.5% glucose were added to the raw acaí liquid and mixed evenly, after which the pH was adjusted to 6.4–6.8 by addition of 1 mg/ml citric acid and calcium carbonate, and the mixture was sterilized at 115°C for 15 min.

#### 2.2.2 Expanded strain culture

Larger scale single‐ and mixed‐culture fermentations were performed to better evaluate the effects of selected *Lactobacillus* strains on the physical, chemical, and volatile aroma components and aroma characteristics of Acaí fermentation liquid. The growth cycle, growth characteristics, and stability of the fermented strains under temperature and pH were screened to screen out the optimal strains and optimal process conditions for the fermentation of Acaí raw materials.

*Leuconostoc mesenteroides*, *Lactobacillus paracasei*, *Lactobacillus plantarum*, *Lactobacillus acidophilus*, *Lactobacillus salivarius*, *Lactobacillus delbrueckii* subsp. *bulgaricus*, *Streptococcus thermophilus*, *Lactobacillus Swiss*, *Lactobacillus fermentans*, and *Pediococcus pentosus* were inoculated into an MRS broth medium at 37°C for 24 h. The number of bacteria reached 10^7^ cfu/ml.

#### 2.2.3 Optimization of the fermentation process

##### Single‐factor experiment

According to the above experimental results, three strains displaying satisfactory performance before and after fermentation were selected for the single‐factor experiment. The inoculation quantity (0.9%, 1.5%, 2.1%, 2.7%, and 3.3%), the proportion of species (the proportion of *Lactobacillus paracasei*, *Leuconostoc mesenteroides*, and *Lactobacillus plantarum* were 0.2:1:1.8, 0.5:1:1.5, 1:1:1, and 1.8:1:0.2), nitrogen source addition amount (1%, 1.5%, 2%, 2.5%, and 3%), fermentation temperature (24, 27, 30, 33, and 37°C), and fermentation time (2, 4, 6, 8, and 10 days) were assessed to explore the influence of related factors on the fermentation process and determine the appropriate fermentation conditions for each single factor. The reference range was provided for subsequent process optimization, and three parallel parameters were set for each factor.

#### 2.2.4 Orthogonal test

The inoculation amount, strain proportion, fermentation time, and nitrogen source addition quantity were selected according to previous studies. Orthogonal experiments with four factors and three levels were performed (Table 1). The oxygen radical absorbance capacity (ORAC) value of the acaí fermentation liquid was considered the evaluation index to determine the influence of various factors and their interaction with the ORAC value of acaí fermentation liquid. The optimal fermentation process conditions were also established. The experimental design and analysis were realized by using SPSS software.

**TABLE 1:** Level table of the orthogonal factors.

Levels	Factors			
	Inoculation amount (%)	Strain proportion	Fermentation time (days)	Nitrogen source addition amount (%)
1	1	0.5:1:1.5	6	1.5
2	1.5	1:1:1	8	2
3	2	1.5:1:0.5	10	2.5

### 2.3 Electronic tongue detection

A 30 ml liquid sample was measured, after which distilled water was added until reaching a constant volume of 60 ml. After stirring for 3 min, 50 g reference liquid was added and centrifuged for 3000 ***g*** for 10 min. The clear liquid was removed and detected using a TS‐5000Z taste analysis system (INSENT Company) (Gupta et al., 2010).

### 2.4 Scanning electron microscopy (SEM)

The freeze‐dried powder samples were smeared on the double‐sided adhesive of the sample tray, followed by nitrogen‐blowing treatment. After the processed samples were vacuumed via SEM, a certain voltage was applied, and the beam spot size was adjusted until the focus was clear. The images were obtained at 500×, and 1000× magnification, respectively, and the differences were observed (Müller et al., 2011).

### 2.5 Determination of the physical and chemical composition

#### 2.5.1 Determination of the basic physical and chemical components

The protein, ash, fat, and moisture content of the COPs were measured using the official AOAC method (AOAC, 2002). The total polyphenol content was determined using an Evolution‐201 Ultraviolet spectrophotometer (Thermo Fisher Technology Co., Ltd.) according to a method described by Abeysinghe et al. (2021).

#### 2.5.2 Determination of the amino acid composition

The amino acid composition was analyzed using an A300 automatic amino acid analyzer (Membrapure, Germany) according to Yang et al. (2007).

#### 2.5.3 Determination of the procyanidin content

The procyanidin content was determined using an LC‐20A HPLC (Shimadzu Company) according to a method described in a previous study Yang et al. (2007).

#### 2.5.4 Determination of the flavonoid content

Here, 50 mg (accurate to 0.001 g) samples were weighed into a centrifuge tube, after which 5 ml methanol was added, shocked for 30 min, ultrasonicated for 30 min, and shocked again for 30 min. The samples were transferred to a 50 ml volumetric flask and fixed with methanol. The samples were filtered into a flask via a 0.22 μm filter membrane at a wavelength of 320 nm on an Ascentis rp‐amide column (3 μm 4.6 × 150 mm). Mobile phase A consisted of pure water, while mobile phase B comprised acetonitrile (Alara et al., 2018).

### 2.6 Determination of the volatile aroma components

The volatile aroma components were determined via Clarus SQ8 gas chromatography–mass spectrometry (GC–MS) with an EI ion source (PerkinElmer), using a WAX ETR 30 m × 0.25 mm × 0.50 μm capillary column. The initial column temperature was 35°C, which was kept constant for 2 min, after which it was raised to 230°C at 4°C/min, where it was maintained for 7 min. The carrier gas used for chromatographic separation was helium (purity 99.999%) at a flow rate of 1 ml/min. The splitless injection was performed and the inlet temperature was set to 240°C. The mass spectrometry was operated at an ion source temperature of 230°C, a transmission line temperature of 240°C, an electron bombardment source of 70 eV, and a scanning range of 30–550 amu. Full SCAN mode (SCAN) was used for qualification, and selective ion SCAN mode (SIM) was used for quantification (Hong et al., 2021).

### 2.7 Chemical composition analysis via liquid chromatography–mass spectrometry (LC–MS)

The freeze‐dried powder sample was dissolved in ultrapure water, filtered, and analyzed via Dinonex Ultimate 3000 UHPLC‐Q Exactive liquid mass spectrometer (Thermo Scientific) equipped with a HESI ion source. The chromatographic separation was achieved on a Waters BEH C18 100 mm × 2.1 mm, 1.7 μm chromatographic column. The injection volume was 5.0 μl. Mobile phase A consisted of 0.1% formic acid water, while mobile phase B comprised acetonitrile. Mass spectrometry analysis was operated in positive (spray voltage of 4.0 kV) and negative ion (spray voltage of 3.2 kV) modes, respectively. The air warping rate and auxiliary gas rate were set to 40 and 10 ml/min, respectively. The capillary temperature was set to 300°C, and S‐lens was 50% (Huang et al., 2021).

### 2.8 Determination of the antioxidant activity

#### 2.8.1 Measurement of the ORAC value

After freeze‐drying, the raw and fermented acaí liquid, about 0.5 g of the freeze‐dried sample, was accurately weighed, mixed with 5 g of sea sand, and thoroughly ground. The uniformly ground sample was transferred to an appropriately sized triangular flask. First, the fat‐soluble portion was extracted with hexane: dichloromethane (1:1, V/V, Hex/Dc): 50 ml Hex/Dc solvent was added and ultrasonically treated at 30°C for 18 min. During the ultrasonic process, the triangular bottle was shaken several times and filtered using filter paper. The extraction was repeated three times. The lipid‐soluble portion was obtained by combining the filtrate. The remaining filtrate residue was then extracted ultrasonically using 50 ml acetone: water: acetic acid (70: 29.5: 0.5, V/V/V, AWA) in the same way and repeated three times. The water‐soluble portion was obtained by combining the filtrate. The water‐soluble and fat‐soluble portions were dried in a rotary evaporator at 45°C and used to determine hydrophilic ORAC (H‐ORAC value) and lipophilic ORAC values (L‐ORAC value), respectively (Wu et al., 2008).

##### H‐ORAC value determination

The experimental group consisted of 25 μl sample liquid and 100 μl fluorescein (a 0.8 μmol/L fluorescent indicator) mixed in a 96‐well plate, to which 75 μl of 150 mmol/L AAPH was added to initiate the reaction. The samples were replaced by 25 μl Trolox standard (6.25, 12.5, 25, 50, 100, 250, and 500 μmol/L) as a positive control (standard curve) and 25 μl phosphoric acid buffer (pH = 7.4, 75 mmol/L) as a blank control. The temperature was maintained at 37°C for 20 min, while the excitation and emission wavelengths of the SpectraMax i3x multifunctional microplate reader (MD Corporation) were set at 485 and 530 nm, respectively. Measurements were taken every 5 min for a total of 150 min. In addition, a control group without AAPH was established. The ORAC value of the samples was calculated according to the ratio of the protected area of the fluorescence decay curve of the samples to the protected area of the fluorescence decay curve of the standard product. The ORAC value of the tested samples was calculated according to the standard curve, and the results were expressed as μmol/g Trolox (Eom et al., 2020; Mario et al., 2019).

##### L‐ORAC value determination

Samples with volumes of 25 and 100 μl of fluorescein (fluorescein, 0.8 μmol/L) were added to a 96‐well plate and maintained at 37°C for 20 min. Then, 75 μl and 187.5 mmol/L of AAPH were added. The excitation and emission wavelengths were set at 485 and 530 nm, respectively, using a fluorescein microplate analyzer. Measurements were performed every 5 min for a total of 180 min. A volume of 2 μl Trolox standard (3.125, 6.25, 12.5, 25, 50, 100, 250, and 500 μmol/L) was used as a positive control to prepare the standard curve. The Trolox standard was prepared with 7% by mass of methylated ‐β ‐cyclodextrin (RMCD) acetone and water at a 1:1 volume ratio. The RMCD was considered a blank control, while AAPH was not added. The ORAC value of the samples was calculated according to the ratio of the protected area of the fluorescence decay curve of the sample to the protected area of the fluorescence decay curve of the standard samples, as well as the standard curve. The results were expressed as μmol/g Trolox (Eom et al., 2020; Mario et al., 2019).

#### 2.8.2 Determination of the DPPH free radical scavenging ability

Here, 100 μl of 0.1 mmol/L DPPH‐anhydrous ethanol liquid and 100 μl sample liquid were added to a 96‐well plate at different concentrations, mixed well, and left to react at room temperature for 30 min, after which the absorbance value A_x_ was determined at a wavelength of 517 nm. The absorbance value A_0_ was measured by mixing 100 μl sample liquid with 100 μl anhydrous ethanol. The sample was replaced with distilled water as a blank control and mixed with 100 μl of 0.1 mmol/L DPPH‐anhydrous ethanol liquid. The absorbance value A_1_ was also measured (Abdulrahman et al., 2019; Sethi et al., 2020; Yang et al., 2010).

$$\text{DPPH free radical scavenging rate}\;\left(\%\right)=\left(1‐\frac{\mathrm{AX}‐A0}{A1}\right)\times 100$$

#### 2.8.3 Determination of the hydroxyl radical scavenging ability

Here, 100 μl sample liquid at different concentrations, 200 μl of 5 mmol/L salicylic acid ethanol liquid, and 200 μl of 5 mmol/L FeSO_4_ liquid were added successively using the salicylic acid method. The reaction in the experimental group (A_X_) was started with 100 μl H_2_O_2_, while the reaction in the control group (A_0_) was started with an equal volume of distilled water. The blank control group (A_1_) contained an equal volume of distilled water. After reacting for 1 h, 200 μl reaction liquid was placed in a 96‐well plate, and the absorbance value was measured at 510 nm (Liu et al., 2015).

$$\text{Hydroxyl radical scavenging rate}\;\left(\%\right)=\left(1‐\frac{\mathrm{AX}‐A0}{A1}\right)\times 100$$

#### 2.8.4 Determination of the ABTS free radical scavenging ability

Here, 100 μl ABTS liquid and 100 μl oxidant liquid were mixed to obtain the ABTS working mother liquid, which was left to stand overnight at room temperature in the dark to yield the ABTS free radical reserve liquid. The ABTS working liquid was diluted 35 times with 0.1 mol/L phosphate buffer (pH 7.4) before use, while the absorbance value at 734 nm was 0.70 ± 0.02, after which 200 μl ABTS working liquid was added to each well. Next, 10 μl of Trolox standard liquid at different concentrations was added to the standard curve detection well, and 10 μl of the sample liquid was added to the sample detection well, mixed gently, and incubated for 6 min at room temperature, after which the absorbance value was determined at 734 nm. The standard curve was drawn using the Trolox liquid concentration as the abscissa and the absorbance as the ordinate. The antioxidant capacity of the final sample was expressed as mmol/g Trolox (Corsetto et al., 2020).

#### 2.8.5 The ferric reducing antioxidant power (FRAP) value

A 7.5 ml volume of TPTZ diluent and 750 μl TPTZ liquid were thoroughly mixed, after which 750 μl TPTZ detection buffer was added to prepare the FRAP working liquid, which was incubated at 37°C for later use. A 180 μl volume of the FRAP working liquid was added to the test well of the 96‐well plate, while 5 μl distilled water was added to the control well, 5 μl FeSO_4_ standard liquid at various concentrations (0.15, 0.3, 0.6, 0.9, 1.2, and 1.5 mmol/L) were added to the test well of the standard curve, and 5 μl sample liquid was added to the test well. Trolox was used as a positive control, and the absorbance value was determined at 593 nm after incubation at 37°C for 5 min. The reducibility of the ferrous iron was expressed via the FeSO_4_ standard liquid concentration in mmol/g FeSO_4_ (Amjadi et al., 2019; Silva et al., 2017).

### 2.9 Statistical analysis

All the tests were done in triplicate (*n* = 3), and statistical analysis was performed by one‐way analysis of variance (anova) using Originpro 8.0 (OriginLab Corp.). Data were presented as means with standard deviation (SD). Statistical significance was set at *p* < .05.

## 3 RESULTS AND DISCUSSION

### 3.1 Electronic tongue analysis

**FIGURE 1:**
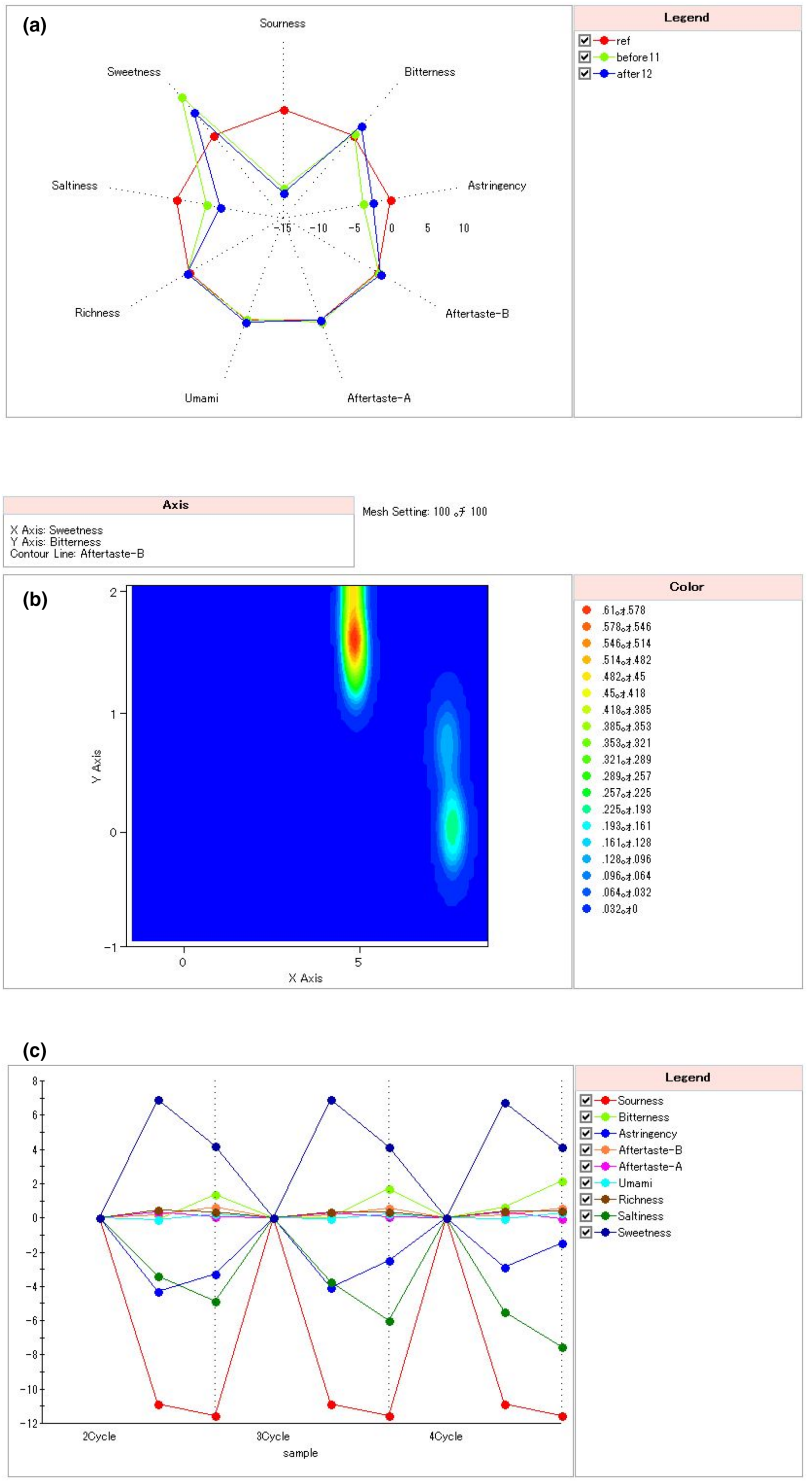
(a) Radar diagram of the taste index of the raw and fermented acaí liquid. (b) An isopotential diagram of the raw and fermented acaí liquid. (c) The stability of the raw and fermented acaí liquid.

The sensors used in the electronic tongue include six taste attributes of bitter, sweet, salty, sour, fresh, and astringent, and two comprehensive attributes of aftertaste‐A and aftertaste‐B. In addition, the difference in taste indicators between each sample can be evaluated by the taste radar chart. Each taste index is marked in the form of a wheel‐shaped coordinate axis. The positive direction of the coordinate axis is the direction of increasing taste value. The scale is the difference in taste that most people can perceive. Figure 1a–c shows the electronic tongue analysis results of the raw and fermented acaí liquid. Certain differences were evident between the sweet and sour tastes of the raw and fermented liquid. After fermentation, the sweet taste of the raw liquid decreased while the sour taste increased, possibly due to the acid produced by lactic acid bacteria metabolizing carbohydrates. This result was consistent with a previous study that used the electronic tongue to characterize the flavor properties of dry‐fermented sausages containing different NaCl substitutes (Chen et al., 2021). The results showed that the sweetness of the product decreased while the sourness increased after fermentation. In addition, the richness of the acaí stock solution and fermentation broth did not change significantly. Richness describes the content of various flavor substances, such as the amino acids formed by protein hydrolysates, which are the source of different flavor substances (Zhao et al., 2016).

### 3.2 SEM analysis

**FIGURE 2:**
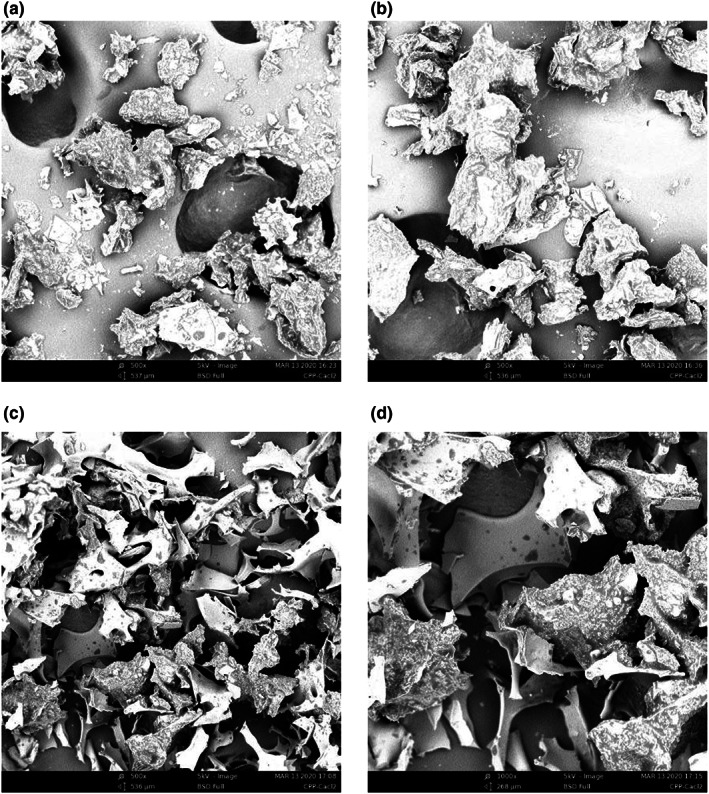
SEM of the lyophilized powder derived from the raw acaí liquid. (a) Magnified 500×. (b) Magnified 1000×. SEM of the lyophilized powder derived from the fermented acaí liquid. (c) Magnified 500×. (d) Magnified 1000×.

SEM was used to examine the micromorphology of the samples before and after acaí fermentation in terms of overall morphology and particle size and observe the freeze‐dried powder of acaí liquid and fermentation liquid at 500× and 1000× magnification, respectively, as shown in Figure 2a–d. Before fermentation, the acaí particles formed irregular blocks while changing significantly after fermentation to exhibit an irregular lamellar bridge skeleton. These microstructural changes may be due to the homogenization, enzymatic hydrolysis, and fermentation of the compound lactic acid bacteria during the fermentation process. Studies have shown that the structures and states of acaí treated with different drying methods vary. IRFD‐dried samples were slightly darker than FD‐dried samples, while those exposed to far‐radiation heating were more compact and harder (Oliveira et al., 2021).

### 3.3 Determination results of the physical and chemical components

#### 3.3.1 The basic physical and chemical component content

The physical and chemical components (moisture, ash, protein, fat, polyphenols, and procyanidins) in the raw and fermented acaí liquid were analyzed. As shown in Table 2, the moisture, ash, protein, fat, polyphenol, and procyanidin levels were 88.99 ± 0.12, 0.02 ± 0.00, 1.30 ± 0.07, 3.60 ± 0.16, 0.82 ± 0.03, and 0.44 ± 0.04 g/100 g, respectively, in the raw acaí liquid and 89.09 ± 0.15, 0.26 ± 0.01, 2.77 ± 0.06, 2.99 ± 0.18, 0.50 ± 0.02, and 0.12 ± 0.01 g/100 g, respectively, in the fermented acaí liquid. After fermentation, the water, ash, and protein levels in the acaí increased, while the total polyphenols and procyanidins decreased.

**TABLE 2:** Physicochemical and amino acid composition of the raw and fermented acaí liquid.

Sample	Moisture (g/100 g)	Ash (g/100 g)	Protein (g/100 g)	Fat (g/100 g)	Polyphenols (g/100 g)	Procyanidins (g/100 g)
Raw acaí liquid	88.99 ± 0.12	0.02 ± 0.00	1.30 ± 0.07	3.60 ± 0.16	0.82 ± 0.03	0.44 ± 0.04
Fermented acaí liquid	89.09 ± 0.15	0.26 ± 0.01	2.77 ± 0.06	2.99 ± 0.18	0.50 ± 0.02	0.12 ± 0.01

Name	Hydrolyzed amino acids		Free amino acids	
	Raw acaí liquid	Fermented acaí liquid	Raw acaí liquid	Fermented acaí liquid
	Content (g/100 g)	Content (g/100 g)	Content (g/100 g)	Content (g/100 g)
Aspartic acid (Asp)	0.108	0.080	0.0017	0.006
Threonine (Thr)	0.061	0.043	0.0022	0.006
Serine (Ser)	0.064	0.081	0.0059	0.006
Glutamic acid (Glu)	0.123	0.614	0.0090	0.018
Glycine (Gly)	0.054	0.061	0.0011	0.005
Alanine (Ala)	0.064	0.078	0.0119	0.015
Valine (Val)	0.063	0.091	0.0028	0.012
Cysteine (Cys)	0.011	0.057	0.0004	0.003
Methionine (Met)	0.003	0.023	0.0005	0.003
Isoleucine (Ile)	0.053	0.060	0.0018	0.009
Leucine (Leu)	0.095	0.109	0.0033	0.028
Tyrosine (Tyr)	0.034	0.037	0.0022	0.006
Phenylalanine (Phe)	0.064	0.082	0.0054	0.010
Histidine (His)	0.025	0.035	0.0014	0.004
Lysine (Lys)	0.072	0.039	0.0016	0.005
Arginine (Arg)	0.052	0.030	0.0012	0.007
Proline (Pro)	0.066	0.207	0.023	0.005
Total	1.012	1.727	0.055	0.148

The protein content in the acaí liquid increased significantly to 2.77 ± 0.06 (g/100 g) after fermentation. This may be due to the enzymes produced by microorganisms during the fermentation process to promote the hydrolysis of proteins with larger molecular weights (Yang et al., 2020). This was consistent with the results of Frias et al. (2008), who used *Lactobacillus plantarum* to ferment soybeans. The small molecular weight proteins also increased, promoting the formation of various flavor substances. Fermentation significantly reduced the total fat, total polyphenol, and anthocyanin content in the acaí. The reduction in the fat content may be due to the fat metabolism by microbial activity. The loss of anthocyanins during fermentation is affected by a variety of factors, for example, during fermentation, anthocyanins may interact with other flavonoids to form more stable anthocyanidins, reducing the polarity and solubility of these compounds (Benito et al., 2011). In addition, anthocyanins and phenolic compounds can react to form complexes, which may also be the reason for the decrease in total polyphenols and anthocyanins (Klopotek et al., 2005).

#### 3.3.2 Amino acid composition

Amino acids can provide a sour, sweet, bitter, or fresh taste and play a role in the flavor of food. Table 2 shows the hydrolyzed amino acid and free amino acid content in the raw and fermented acaí liquid. After fermentation, the acaí amino acid content increased, which was consistent with the protein determination results. In the case of hydrolyzed amino acids, the contents of glutamic acid, aspartic acid, leucine, lysine, and proline were higher in the raw acaí liquid, while the fermentation liquid displayed higher glutamic acid, proline, leucine, valine, and phenylalanine levels. In terms of free amino acids, the contents of alanine, glutamic acid, serine, phenylalanine, and leucine were higher in the raw acaí liquid, while the fermentation liquid exhibited higher leucine, glutamic acid, alanine, valine, and phenylalanine levels. These amino acids provided the acaí products with a sweet and sour taste.

After fermentation, the total amino acids in the acaí products increased from 1.012 to 1.727 (g/100 g). The highest nonessential amino acid content in the acaí stock solution was glutamic acid, followed by aspartic acid, lysine, proline, serine, alanine, and arginine. The highest content of essential amino acids is leucine, followed by lysine, phenylalanine, valine, valine, threonine, and the lowest is methionine. Yang et al. (2020) used fermentation and auxiliary enzymolysis to improve the quality of soybean meal protein and the degradation of allergens. The results presented by the amino acid map were consistent with those of the acaí stock solution. This indicates that acaí is a good source of amino acids and can be used for essential amino acid supplementation. After fermentation, the content of most amino acids increased significantly, especially that of glutamic acid, increasing the umami and sour taste of the acaí products, which was consistent with the electronic tongue analysis results. The increased concentrations of certain amino acids can be attributed to the microbial metabolic activity during acaí fermentation (Yang et al., 2020).

Research has shown that the amino acid composition of seaweed can be changed by microbial fermentation treatment (Norakma et al., 2022). Therefore, fermentation can increase the amino acid concentration in acaí products. Studies have shown that amino acids such as tyrosine, histidine, cysteine, and methionine display antioxidant activity. Histidine exhibits strong free radical scavenging activity due to the decomposition of its imidazole ring, while cysteine acids display strong reducibility due to the presence of sulfhydryl groups (Virtanen et al., 2007). The antioxidant activity of fermented acaí in this study may be due to the presence of these amino acids.

#### 3.3.3 Flavonoid content

**FIGURE 3:**
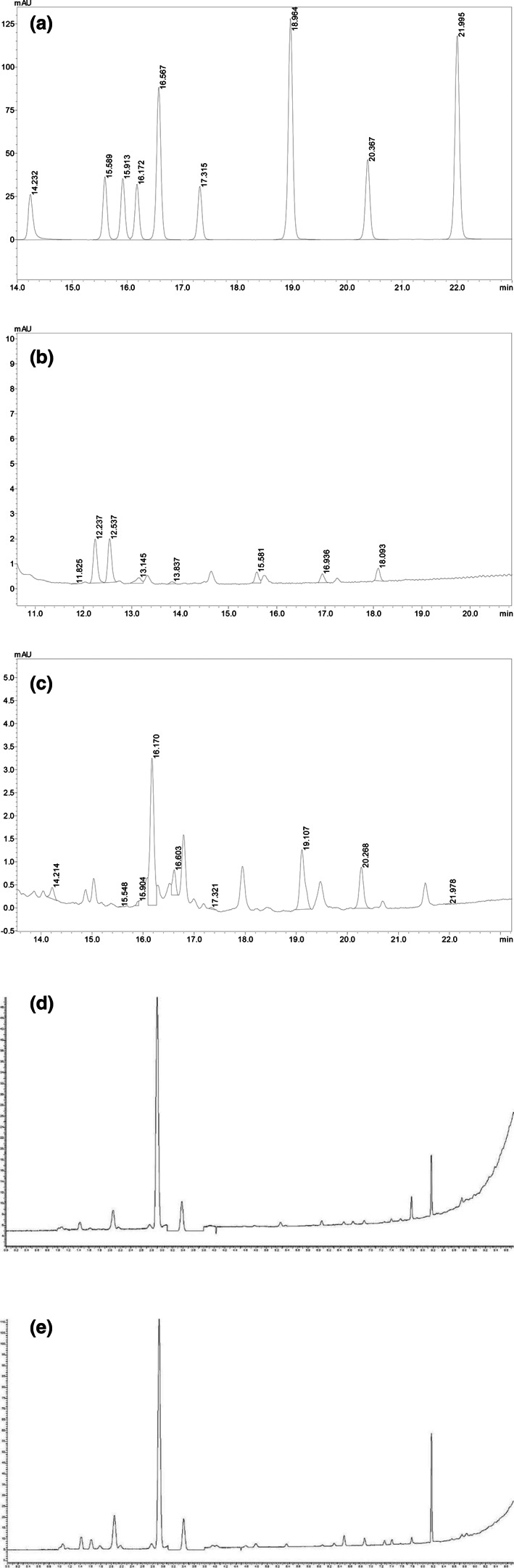
(a) HPLC of the flavonoid standard. (b) A liquid chromatographic diagram of the acaí liquid. (c) Liquid chromatography of the fermented acaí liquid. (d) A gas chromatogram of the acaí liquid. (e) A chromatogram of the fermented acaí liquid.

The liquid chromatographic diagram of the flavonoid standard is shown in Figure 3a. The liquid chromatographic diagrams of the nine flavonoids in the raw and fermented acaí liquid are shown in Figure 3b,c, while the flavonoid content results are shown in Table 3. The total content of the nine flavonoids in the acaí decreased after fermentation. The 4′OH‐nobiletin, 5′OH‐tan levels, 4′OH‐tan, and nobiletin in the raw acaí liquid were higher, while that of sinensetin, tangeretin, 3′,4′OH‐nobiletin, and 5′OH‐nobiletin increased after fermentation. The reason for the decrease in the total content of flavonoids may be as mentioned above. During fermentation, flavonoids interact with anthocyanins to form more stable anthocyanidins, reducing their content (Benito et al., 2011).

**TABLE 3:** Contents of flavonoids and volatile components in the raw and fermented acaí liquid.

Name	Raw acaí liquid	Fermented acaí liquid
	Content (g/100 g)	Content (g/100 g)
3′,4′OH‐Nobiletin	0.090	0.197
3′OH‐Nobiletin	0.011	0.009
4′OH‐Nobiletin	0.227	0.020
Sinensetin	0.203	0.281
4′OH‐Tan	0.053	0.044
Nobiletin	0.029	0.013
Tangeretin	0.153	0.208
5′OH‐Nobiletin	0.122	0.155
5′OH‐Tan	0.169	0.082
Total	1.058	1.007
	Content (mg/L)	Content (mg/L)
Acetaldehyde	0.24	0.94
Dimethyl sulfur	0	0.07
Ethyl formate	0	0.09
Ethyl acetate	0.07	0.22
N‐propanol	0.19	1.33
Isobutanol	0	0.38
Isoamyl alcohol	0.14	1.20
Total	0.64	4.23

### 3.4 Analysis of the volatile aroma components

The chromatograms of the raw and fermented acaí liquid are shown in Figure 3d,e. The results of the volatile aroma components in the two acaí products are shown in Table 3. Four volatile aroma components were identified in the raw liquid, which was represented from high to low by acetaldehyde, n‐propanol, isoamyl alcohol, and ethyl acetate. Seven volatile aroma components were produced in fermentation liquid, which was represented from high to low by n‐propanol, isoamyl alcohol, acetaldehyde, isobutanol, ethyl acetate, ethyl formate, and dimethyl sulfur. The contents of all volatile components increased after fermentation, and three new volatile components were added, namely, isobutanol, ethyl formate, and dimethyl sulfur. Therefore, the fermentation process can improve the flavor of acaí products.

The increase in the type and content of the volatile components in the acaí products after fermentation may result from microbial metabolic activity. This result was consistent with that of Bleve et al. (2015), who found that the volatile components of table olives increased after fermentation. They concluded that the volatile components in table olives result from the metabolic interaction between yeast and bacteria. This activity significantly affected the chemical composition, resulting in a more complex aroma. Yan et al. (2018) found that the volatile components in strawberry‐flavored products mainly comprised esters and alcohols, among which methyl butyrate and ethyl butyrate were the key flavor compounds.

### 3.5 Chemical composition identification via LC–MS

**FIGURE 4:**
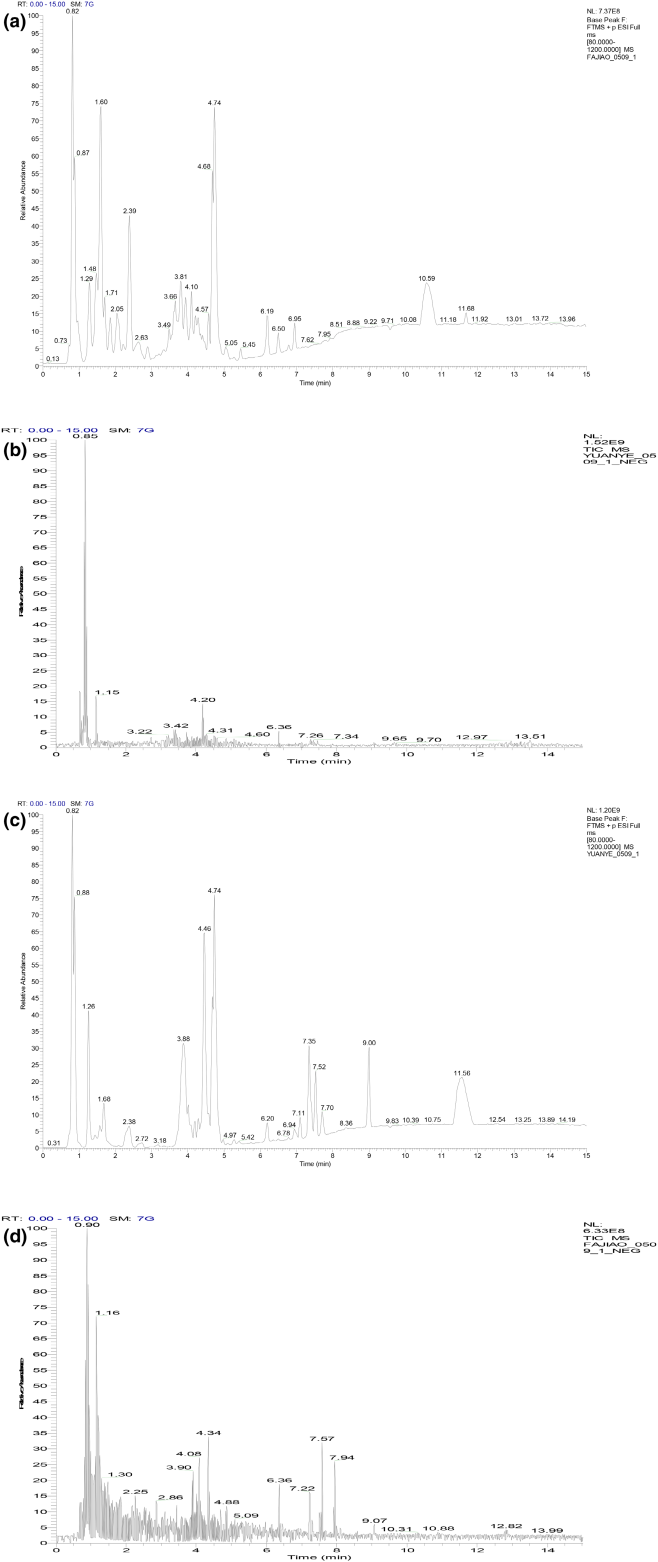
(a,b) The ion flow and mass spectrometry of the acaí liquid. (c,d) The ion flow and mass spectrometry of the acaí fermentation liquid extraction.

The chemical composition of acaí before and after fermentation was examined via LC–MS. The results are shown in Figure 4a–d and Table 4, identifying 98 chemical components, including flavonoids, organic acids, anthocyanins, and other chemical components. According to Table 4, the type and content of the chemical components changed before and after fermentation. During the fermentation process, the bacteria can produce a variety of enzymes and other primary and secondary metabolites to catabolize the acaí, which can degrade many large molecular compounds that cannot be directly absorbed and utilized by the human body into small molecular compounds, producing new active substances and functions.

**TABLE 4:** Identification and analysis of the chemical components in the raw and fermented acaí liquid.

Name	Acaí raw liquid	Acaí fermentation liquid
	Content (%)	Content (%)
Eupatilin	0.2368	2.0057
Poricoic acid A	0.0010	1.5668
L(−)‐Carnitine	0.3332	1.3732
Astragalin	5.6307	1.2856
Ethyl caffeate	0.3334	1.2704
Methyl 4‐hydroxy‐3‐methoxycinnamate	0.3334	1.2704
Blinin	0.1756	1.1949
7‐O‐Ethylmorroniside	0.0279	1.1047
Pectolinarigenin	0.0850	0.9546
L‐Tryptophan	0.1176	0.9428
Forskolin	0.0061	0.8749
1‐O‐Acetylbritannilactone	0.0812	0.8682
Diosbulbin B	0.1214	0.7969
Atractyloside A	0.1120	0.7624
Taurochenodeoxycholic Acid	0.0042	0.7254
Praeruptorin A	0.2380	0.6921
p‐Coumaric acid	0.0577	0.6444
Hosenkoside K	–	0.5804
Cytosine	0.0913	0.5804
L‐Glutamic acid	0.1000	0.5765
Desoxyrhaponticin	0.0461	0.5661
Stachydrine	1.0178	0.5499
4’‐O‐Glucosylvitexin	4.0139	0.5264
Pyrogallol	0.1122	0.5224
Picroside I	0.1253	0.4707
Spiculisporic Acid	0.0012	0.4572
Jaceosidin	0.1084	0.4556
Isovitexin	0.2198	0.4469
Diffractaic Acid	0.1195	0.4375
Dihydrocapsaicin	0.0181	0.4373
Sinensetin	0.1633	0.4347
Gambogenic acid	0.0064	0.4088
Steviol	0.0466	0.3776
Mesaconitine	–	0.3680
Curcumol	0.0533	0.3569
S‐(−)‐Carbidopa	0.0143	0.3553
Ligustrazine HCl	0.0006	0.3538
Artemisinic acid	0.1039	0.3442
Kushenol F	0.0004	0.3378
Nicotinic acid	0.4965	0.3336
3‐Isomangostin	0.0014	0.3281
Sauchinone	0.0384	0.3269
Germacrone	0.1116	0.3199
Cyasterone	0.0011	0.3174
Isoalantolactone	0.0534	0.3168
Ailanthone	0.0245	0.3062
Rosavin	0.0824	0.3050
Eurycomalactone	0.0596	0.3009
Anisic aldehyde	0.1082	0.2970
Diosmetin‐7‐O‐beta‐D‐glucopyranoside	0.2700	0.2929
Artesunate	0.0193	0.2921
Isocorynoxeine	0.0003	0.2918
Isopropyl 4‐Hydroxybenzoate	0.0571	0.2860
Isoeugenol acetate	0.1180	0.2727
Ethyl 4‐methoxycinnamate	0.0599	0.2727
Columbin	0.0478	0.2713
Pristimerin	0.0057	0.2577
L‐Theanine	0.0142	0.2572
8‐Prenylnaringenin	0.0981	0.2533
Epimagnolin B	0.0004	0.2518
Alnustone	0.0141	0.2506
Methyl 4‐hydroxycinnamate	0.0859	0.2502
Loganic acid	0.1119	0.2452
Dehydroandrographolide	0.0172	0.2406
Aucubin	0.0290	0.2285
3‐Butylidenephthalide	0.0643	0.2282
2‐Adamantanone	0.0564	0.2280
Perillene	0.0564	0.2280
Vincristine	0.0240	0.2189
Cinnamic acid	0.0293	0.2154
Coumarin	0.0983	0.2023
Qingyangshengenin	0.0018	0.2022
4‐Methylumbelliferone	0.1301	0.1999
Isopropylidenylacetyl‐marmesin	0.0927	0.1970
Arglabin	0.0572	0.1953
Hispidulin	0.0279	0.1936
Hypaconitine	–	0.1919
Abietic Acid	0.0584	0.1909
Sarracenin	0.1077	0.1839
Quercetin	0.0338	0.1783
Adenine	2.2768	0.1747
Pseudolaric Acid B	0.4173	0.1622
Octyl gallate	0.0549	0.1616
Eupalinolide A	1.1495	0.1539
5‐Hydroxy‐1‐tetralone	0.1059	0.1431
Dehydrodiisoeugenol	0.2372	0.1267
Alpha‐Linolenic acid	0.0820	0.1030
Nicotinamide	0.1635	0.0830
Jasminoside B	0.2230	0.0767
Luteolin	0.6835	0.0713
Calcium pantothenate	0.1271	0.0681
Eriodictyol	0.4473	0.0479
Diosmin	0.7348	0.0251
Spinosin	0.7348	0.0251
Leucoside	0.1506	0.0164
Sciadopitysin	0.1466	0.0164
Vitexin rhamnoside	0.1368	0.0132
Taxifolin	0.2077	0.0132

### 3.6 The antioxidant results of the acaí before and after fermentation

#### 3.6.1 ORAC value

##### H‐ORAC value

**FIGURE 5:**
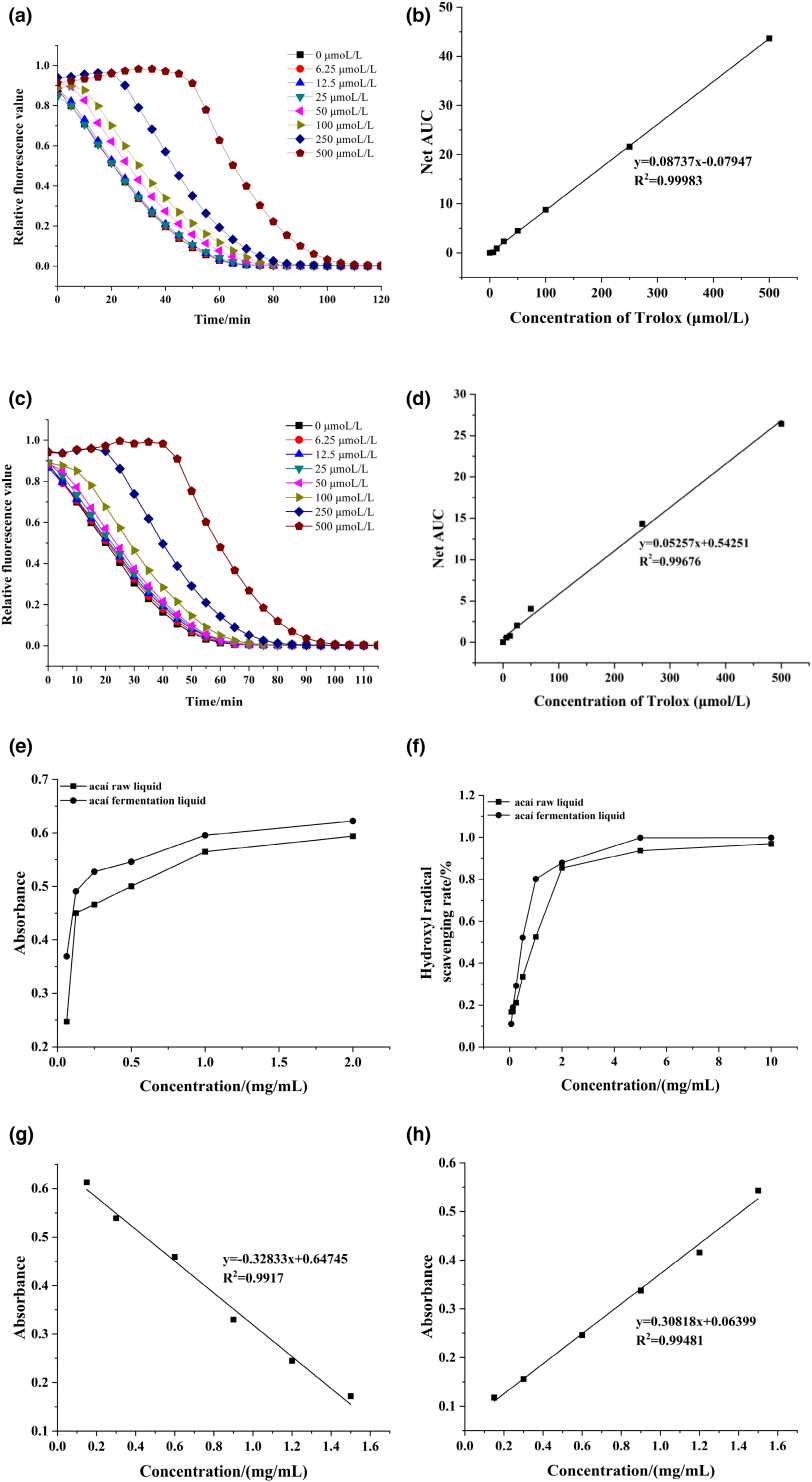
(a) The dynamic fluorescence attenuation curves of Trolox at different concentrations. (b) The Trolox standard curve. (c) The dynamic fluorescence attenuation curves of Trolox at different concentrations. (d) The Trolox standard curve. (e) The DPPH free radical scavenging capacity of the two acaí samples. (f) The hydroxyl radical scavenging capacity of the two acaí samples. (g) The Trolox standard curve of ABTS. (h) The FeSO_4_ standard curve.

Oxygen radical absorbance capacity (ORAC) refers to an antioxidant capacity index, which is also known as antioxidant radical scavenging capacity or antioxidant capacity index measurement. ORAC is an evaluation method used in the field of antioxidant research. The H‐ORAC value was used to evaluate the ORAC value of the water‐soluble antioxidants. The dynamic fluorescence attenuation and standard curves of different Trolox concentrations are shown in Figure 5a,b, *y* = 0.08737*x*−0.07947, *R* ^2^ = .99983. According to the standard curve, the H‐ORAC values of the freeze‐dried powder derived from the raw and fermented acaí liquid were 453.64 ± 24.28 μmol/g Trolox and 708.77 ± 12.46 μmol/g Trolox, respectively. After fermentation, the H‐ORAC value of the acaí increased by 56.24%. The water‐soluble antioxidant capacity of the fermented acaí product was similar to that of sage spice (987.14 μmol/g Trolox), 30 times that of wolfberry (31.70 μmol/g Trolox), and 60 times higher than that of red grapes (16.40 μmol/g Trolox) (Buratto et al., 2021).

##### L‐ORAC value

RMCD was used to measure the L‐ORAC value and evaluate the ORAC of fat‐soluble substances by incorporating them into the cavity to increase water solubility. The dynamic fluorescence attenuation curves and standard curves of the different Trolox concentrations are shown in Figure 5c,d, *y* = 0.05257*x* + 0.54251, *R* ^2^ = .99676. According to the standard curve, the L‐ORAC values of the raw and fermented acaí liquid were 141.80 ± 10.40 μmol/g Trolox and 168.28 ± 13.18 μmol/g Trolox, respectively. After fermentation, the L‐ORAC value of the acaí samples increased by 26.48%. The ORAC values (H‐ORAC value + L‐ORAC value) of the acaí samples before and after fermentation were 595.45 ± 30.71 μmol/g Trolox and 877.05 ± 20.58 μmol/g Trolox, respectively, while the ORAC value increased by 47.29% after fermentation.

After fermentation, the ORAC of the acaí samples improved significantly. This may be related to higher amino acid concentrations after the fermentation. For example, histidine exhibits strong free radical scavenging activity due to the decomposition of its imidazole ring. The peroxyl radical scavenging capacity determined via ORAC was in the range of acaí results reported by other authors (Kang et al., 2011). Some studies have found that heat treatment can reduce the free radical scavenging ability of acaí juice (da Silveira et al., 2019), while Tadapaneni et al. (2014) found that the free radical scavenging ability of heat‐treated strawberry juice and strawberry milk‐based beverages lost up to 40%. Therefore, the fermentation process of acaí products can be used as a reliable method for further processing, while it is suggested that consuming fermented acaí products may provide antioxidative protection to promote human health.

#### 3.6.2 DPPH radical scavenging ability

DPPH free radical scavenging ability is an important index to evaluate the antioxidant capacity of substances based on electron transfer and charge neutralization principles. A DPPH free radical is a stable N ion radical in an organic environment with a maximum absorption peak at 517 nm (Ganguly et al., 2020). When a radical scavenger is present in the system, the single electron of the DPPH radical is neutralized by the scavenger, and the color of the liquid changes from purple to light yellow or colorless, directly indicating a decrease in the absorbance value. As shown in Figure 5e, the two acaí products displayed significant DPPH radical scavenging ability in a range of 0.0625–2.00 mg/ml, while the scavenging rate was dose dependent with the mass concentration. The DPPH free radical scavenging ability of the acaí fermentation liquid was stronger than that of the raw acaí liquid. The DPPH radical scavenging rate of the acaí samples before and after fermentation was compared, yielding IC_50_ values of 0.58 and 0.16 mg/ml, respectively. The acaí scavenging ability improved after fermentation.

Previous research has shown that acaí displays strong antioxidant activity, and compared with the results of this study, the hydroalcoholic extract from acaí seeds exhibits higher antioxidant activity, corresponding to the EC50 of water extract at 8.8 μg/ml (DPPH assay) (Barros et al., 2015). Martins et al. (2020) studied the chemical properties and antioxidant and antibacterial activity of acaí extracts containing type A and type B proanthocyanidins and found that acaí seed extract and its components provided protection against oxidative stress via hydrogen peroxide.

#### 3.6.3 Hydroxyl radical scavenging ability

A hydroxyl radical is an extremely active free radical formed in a biological system that can cause lipid peroxidation of cell membranes and is extremely harmful to the human body. The hydroxyl radical scavenging capacity of the two acaí samples is shown in Figure 5f. The hydroxyl radical scavenging ability of the acaí samples showed an obvious dose‐dependent relationship and was positively correlated with the experimental concentration range. The acaí samples displayed distinct hydroxyl radical scavenging ability, while the scavenging rate increased at a higher mass concentration, showing a significant dose‐dependent relationship. The hydroxyl radical scavenging ability of acaí fermentation liquid was higher than that of raw acaí liquid. After fermentation, the IC_50_ value of the acaí hydroxyl radical scavenging rate was 0.97 and 0.49 mg/ml, respectively, indicating an improved scavenging ability. The hydroxyl radical scavenging ability is related to its capacity as a hydrogen donor to reduce free radicals and stop the free radicals chain reaction.

Studies have shown that the phenolic compound content is proportional to increased hydroxyl radical levels. Márquez et al. (2019) studied the generation of hydroxyl radicals in white wine and their relationship with phenolic compounds and concluded that the changes in the hydroxyl radicals were related to the phenolic constituents in the wine. Therefore, the reason for the improvement in the hydroxyl radical scavenging ability of the acaí products after fermentation may be due to a reduction in the total phenolic content, decreasing the hydroxyl radicals, and increasing the reducing amino acid content.

#### 3.6.4 ABTS free radical scavenging ability

ABTS was used as a color initiator in this method. The principle was that after adding an oxidant, the ABTS reagent could generate stable blue‐green radical ABTS^+^ in the liquid, yielding maximum absorption at 734 nm. The addition of antioxidant substances neutralized the ABTS^+^ charge, causing the color to become lighter and decreasing the absorbance value. The decreasing trend reflected the strength of the antioxidant of the detected substance. Figure 5g shows the ABTS curve drawn based on the Trolox standards. Calculations showed that the ABTS radical scavenging ability of the freeze‐dried powder derived from the raw and fermented acaí liquid was 0.44 ± 0.03 mmol/g Trolox and 0.45 ± 0.08 mmol/g Trolox, respectively. The acaí fermentation liquid displayed a better scavenging ability than the raw acaí liquid. After fermentation, the ABTS radical scavenging rate of the acaí was slightly higher.

The ABTS method determines the ability of antioxidants to quench ABTS^+^ free radicals via electron transfer reactions. Studies have shown that the phenolic compounds in the acaí are excellent electron donors and can quench ABTS^+^ free radicals (Garzón et al., 2017). Therefore, the fermented acaí product displayed a satisfactory ABTS^+^ free radical scavenging rate.

#### 3.6.5 FRAP value

FRAP is a rapid, simple indicator of antioxidant capacity and can be used to determine the reducing capacity of substances. The standard curve of FeSO_4_ is shown in Figure 5h, which was used to calculate the FRAP values of the two acaí samples. The FRAP values of freeze‐dried powder derived from the raw and fermented acaí liquid were 0.43 ± 0.01 and 0.55 ± 0.02 mmol/g FeSO_4_, respectively. A higher FRAP value typically indicates a stronger antioxidant capacity. The FRAP value of the acaí fermentation liquid was higher than the raw liquid.

Although studies have indicated that phenolic compounds can reduce Fe(III) to Fe(II), this has nothing to do with the generation of hydroxyl radicals, which is a relatively complicated process (Márquez et al., 2019). Therefore, the increased FRAP value of the acaí products after fermentation may be due to a higher total phenolic content. Phenolic compounds interact with Fe(III) in different ways, depending on their chemical structures (Perron & Brumaghim, 2009). Santos et al. (2021) studied the in vitro antioxidant activity of fermented passion fruit alcoholic beverages, revealing higher antioxidant activity than in natural passion fruit beverages, which was due to the increased total phenolic content.

## 4 DISCUSSION

By detecting the basic physical, chemical, and microbial indexes of raw acaí liquid, this study examined the acaí fermentation process via strain screening and four‐factor and three‐level orthogonal tests while establishing methods to determine the H‐ORAC and L‐ORAC values. The ORAC values of the raw and fermented liquid were determined in different fermentation conditions. Furthermore, the optimal fermentation conditions were identified, and the actual fermentation degree was determined. Next, the antioxidant activity of the freeze‐dried powder derived from the raw and fermented acaí liquid was evaluated in terms of eight aspects, while the two samples were analyzed via SEM and electronic tongue detection. The physical and chemical components (water, ash, protein, fat, polyphenols, amino acids, γ‐aminobutyric acid, procyanidins, organic acids, and flavonoids) and volatile aroma components were analyzed and identified.

The fermentation process showed that the order of the influencing factors on the ORAC value of acaí after fermentation via compound lactic acid bacteria was as follows: inoculation amount > nitrogen source addition amount > fermentation time > strain proportion. The optimal fermentation condition combination was A1B1C1D3, that is, the inoculation amount was 1%. The parameters included a strain ratio of *Lactobacillus paracasei*: *Leuconostoc mesenteroides*: *Lactobacillus plantarum* = 0.5:1:1.5, a fermentation time of 6 days, and a nitrogen source supplemental level of 2.5%. The results verified that in optimal conditions, the ORAC value of the fermentation liquid reached the highest value of 273.28 ± 6.55 μmol/L Trolox, which was 55.85% higher than the raw liquid. Although there was no peculiar smell, a specific acaí flavor was present.

The antioxidant activity evaluation results showed that the ORAC value (H‐ORAC value+L‐ORAC value) of the lyophilized powder derived from the raw and fermented acaí liquid were 595.45 ± 30.71 μmol/g Trolox and 877.05 ± 20.58 μmol/g Trolox, respectively, and increased by 47.29% after fermentation. In addition, the FRAP value of the acaí, as well as its scavenging ability of DPPH, hydroxyl, and ABTS free radicals, increased after fermentation.

By comparing the changes in microstructure and physicochemical properties of acaí before and after fermentation treatment, it can be concluded that after fermentation treatment, the microstructure, basic physicochemical composition, amino acid composition, γ‐aminobutyric acid, a variety of volatile components, and so on have changed; furthermore, it is worth noting that the contents of γ‐aminobutyric acid, lactic acid, and a variety of volatile components have significantly increased. Therefore, fermentation treatment can significantly improve the nutritional value and flavor of the acaí. The content of the volatile and various chemical components increased, while fermentation significantly improved the nutritional value and flavor of the acaí.

In summary, the DPPH, ABTS, hydroxyl radical scavenging ability, ORAC, and FRAP data results provide evidence that fermented acaí products display strong antioxidant capacity and can interact with different free radicals. These products may present food health and biomedical applications for reducing oxidative stress in the body. In addition, the antioxidant capacity of any food sample results from the synergistic effect of a mixture of compounds, including phenols, carotenoids, and vitamins C and E. Although the vitamin content of acaí is relatively low, polyphenols play a substantial role in its antioxidant properties (Maria do Socorro et al., 2011).

## 5 CONCLUSION

In this study, acaí fermentation liquid was prepared by fermentation technology. The antioxidant activities of acaí stock solution and fermentation liquid were evaluated from eight aspects. At the same time, the samples before and after fermentation were analyzed by scanning electron microscopy, electronic tongue detection, physical and chemical components, volatile aroma components, and so on. The results showed that acaí fermentation liquid had a strong antioxidant capacity after fermentation treatment, and the fermentation treatment changed the microstructure and flavor of acaí and increased the content of volatile components and various chemical components. In conclusion, fermentation treatment of acaí can improve its quality and nutritional value, which provides a theoretical basis for the comprehensive utilization of acaí.

## CONFLICT OF INTEREST STATEMENT

The authors declare no conflict of interest.

## Data Availability

The data that support the findings of this study are available on request from the corresponding author upon reasonable request.
